# Development and Validation of the Youth Career Development Competency Scale: A Study Based on Hong Kong Youth

**DOI:** 10.3390/ijerph182312494

**Published:** 2021-11-27

**Authors:** Steven Sek-yum Ngai, Lin Wang, Chau-kiu Cheung, Jianhong Mo, Yuen-hang Ng, Pinqiao Wang

**Affiliations:** 1 Department of Social Work, The Chinese University of Hong Kong, Hong Kong, China; linwang@cuhk.edu.hk (L.W.); lannymo@cuhk.edu.hk (J.M.); yhng@swk.cuhk.edu.hk (Y.-h.N.); keirapqwang@cuhk.edu.hk (P.W.); 2 Department of Social and Behavioral Sciences, City University of Hong Kong, Hong Kong, China; ssjacky@cityu.edu.hk

**Keywords:** career development competence, youth, school-to-work transition, scale validation, career guidance

## Abstract

The challenging labor market conditions concomitant with economic globalization and advanced technology have made youth career development competency (YCDC)—young people’s ability to navigate transitions through education into productive and meaningful employment—especially important. The present study aims to develop a holistic instrument to measure YCDC in Hong Kong, which has rarely been investigated in past studies. The sample consisted of 682 youths aged 15–29 years (387 male, mean age = 19.5 years) in Hong Kong. Exploratory factor analysis of the 17-item YCDC scale resulted in four competence factors—engagement, self-understanding, career and pathway exploration, and planning and career management—which accounted for 78.95% of the total variance. The final confirmatory factor analysis results indicated good model fit (CFI = 0.96, TLI = 0.95, RMSEA = 0.06, 90% CI (0.05, 0.07), SRMR = 0.03) and good factor loadings (0.78–0.91). Moreover, the results demonstrated a satisfactory internal consistency of subscales (0.89–0.93). Subgroup consistency across subsamples categorized by gender, age, and years of residence in Hong Kong was also demonstrated. In addition, correlations between the YCDC scale and subscales with other career-related and psychosocial outcomes (i.e., career outcome expectancy, career adaptability, civic engagement, social contribution, and social integration) showed good concurrent validity. The results indicated that the YCDC scale is a valid and reliable tool for measuring career development competence among youth in the Hong Kong context. Its development sheds light on how career professionals can holistically assess young people’s navigation competence during their school-to-work transitions.

## 1. Introduction

Labor markets around the world have shifted from manufacturing to service industries owing to economic globalization and advanced technology, leading to an unpredictable job environment characterized by keen competition and job transformations [1]. Such transformations have yielded considerable effects on young people experiencing the school-to-work transition, as they have to move from a previously clearly defined pathway into a less controlled and predictable terrain [2,3]. Moreover, the availability of multiple pathways in the transformed labor market complicates the entire process of school-to-work transitions, making it more challenging for young people to make a career decision and achieve a productive livelihood [4]. Consequently, today’s youth at the start of their career journeys are at a greater risk of finding only temporary employment as well as experiencing unsatisfactory employment, poor work conditions, and high levels of career discontinuity and underemployment [5,6]. These challenges in their school-to-work transitions may further constrain the formulation and pursuit of educational and career goals, often resulting in a lack of social mobility and a series of social, emotional, and behavioral problems, such as social withdrawal, low self-confidence, hopelessness, passivity, and criminal behavior [7,8,9].

Accordingly, enhancing youth career development competency (YCDC)—young people’s ability to navigate transitions through education into productive and meaningful employment—is crucial for youth to keep up with an increasingly dynamic and changing work environment, adjust to more complex careers, and take responsibilities for performing their job duties and managing their careers [10,11,12,13]. However, research on the development and validation of YCDC instruments for people 15–29 years of age is scarce. Therefore, the present study aims to develop a holistic measurement instrument that can be used among youth aged 15–29 in Hong Kong.

### 1.1. Model Development: Indicators of Career Development Competence among Youth

School-to-work transition refers to the period during which graduates enter the labor market and find work [14]. It includes the experiences and outcomes of an individual’s movements between educational and labor market statuses [15]. Previous research illustrates that the school-to-work transition has an impact on graduates’ labor market participation and status, education–job match, and career mobility [6,14]. Moreover, as mentioned in the introduction, during school-to-work transitions, youth have been impeded in pursuing their career and life goals due to challenges and uncertainties associated with economic globalization and rapid technological advancements [4,5,6]. Given the importance and difficulty of the school-to-work transition, enabling young people to develop competences to navigate this transition is paramount. Among the existing literature on youth career development, the sense of personal agency, which refers to the feeling of control over actions and their consequences, is highlighted as an indispensable element for youth’s career and life development [4]. Young people are expected to cultivate self-awareness and practice self-reflection, which can facilitate their building of career identity and aspiration [10,16]. Furthermore, abilities in planning, implementing, and managing one’s career decision-making are critical, as these competences not only equip youth to navigate multiple pathways and attain smooth school-to-work transitions but also prepare them to overcome obstacles and challenges to achieve desired career goals in their future life journey [4,17,18].

Given the preceding considerations, YCDC is essential, as it represents a strong sense of personal agency and commitment among youth to become active path navigators and change agents in their career pathways. As such, the purpose of strengthening YCDC is to help young people develop meaningful involvements in career-related exposure and social-connection experience as well as enhance their ability to navigate opportunity structures. In this sense, developing a valid YCDC measurement instrument is a prerequisite to effectively enable young people to identify their career development competences, approach labor market entry, and imagine career progression in an informed and confident manner [16,19,20].

In reviewing the available literature on the development of YCDC measurement instruments, previous studies have attached importance to self-understanding, which refers to young people’s ability to comprehend their hopes and dreams about work and life, engage in self-reflection and self-enhancement activities, and connect self-knowledge with pathway alternatives (e.g., further studies, career options, and serious leisure activities) as one of the core career development competences for career success [10,11,21,22]. Moreover, career and pathway exploration represent young people’s ability to explore multiple pathways and forms of work and careers, compare pathway alternatives and prioritize them, and set career and life goals [22]. Studies that focus on lifelong learning and employee employability regard career and pathway exploration as another core career development competence for career success [11,23,24]. In addition, planning and career management refers to young people’s ability to seek support and opportunities, overcome obstacles to achieve career and life goals, and manage career transitions and developments. Prior studies have emphasized the importance of deliberate actions undertaken by individuals to realize their career goals [17]. Planning and career management, thus, have been highlighted as crucial career-development competences [17,25]. Based on previous studies, measuring abilities in self-understanding, career and pathway exploration, and planning and career management are necessary components in the development of a YCDC measurement instrument.

Furthermore, it is noteworthy that previous studies of YCDC measurement tools have put less emphasis on an important yet often overlooked factor—engagement [10,11,26,27]. According to work psychologists [28], engagement represents young people’s high levels of activation and positive involvement in activities or tasks, which are observable in the domains of affect, behavior, and cognition. Improving engagement is crucial for young people because it helps to draw out, ignite, or re-ignite the motivation and passion within them through involvement in new meaningful social and career experiences [22]. In this connection, engagement can be understood as young people’s ability to get involved in new experiences, extend their connection with others and the community, and maintain an interest in participating in career and life planning activities. As a core career development competence, engagement is crucial in predicting career performance [29]. Therefore, further development of YCDC scales must incorporate engagement as an important dimension to bridge the existing knowledge gap.

### 1.2. YCDC in Hong Kong Chinese Context

The unique sociocultural characteristics of Hong Kong may influence YCDC in the city. In 2020, under the impact of the COVID-19 pandemic, the rate of unemployment among young people aged 15–19 and 20–29 in Hong Kong was 18.9% and 9.9%, respectively, compared to the overall 7.0% unemployment rate of the total population [30]. This figure highlights the issue of exclusion from activities required for the transition to adulthood [31]. Facing this stress, attention should be refocused on enhancing YCDC, which is crucial to help young people, especially those experiencing school-to-work transitions, adapt to a challenging work environment [3]. However, many schools do not view school-to-work transitions as a “burning” educational issue in the agenda of school development. Most provisions related to career and life development are confined to providing students with up-to-date information regarding further study opportunities within Hong Kong and abroad. Similarly, Chinese parents consider college or further education as the most promising choice for their children [2]. Consequently, most young people in Hong Kong have a firm sense of direction on further study, but only about half of them have a sense of direction on their future careers [32]. As a result, young people’s understanding of their career development competence and the labor market is overly narrow and distorted due to this sociocultural background [16]. In the face of the influence of the pandemic and the high unemployment rate, young people need to improve their career development competence to successfully navigate their transitions through education into employment.

In addition, Chinese society emphasizes a collectivistic culture and family orientation [33], which may influence YCDC in Hong Kong, as social expectations and obligations are given priority over individual preferences [34]. This notion exerts an impact on one’s career development competence [35]. Likewise, family orientation influences young people’s career exploration [36,37]. For example, with the expectations of developing a desirable social image and preserving respect from others, Chinese parents value academic and career achievement [38]. These expectations exert an impact on young people’s educational and occupational aspirations [39], which further influence how young people make decisions about education and career pathways [40]. In this sense, enhancing YCDC is crucial in providing young people the opportunity to think about, explore, and experience the world of work to explore multiple pathways and develop a greater capacity to aspire [16]. Therefore, a rigorous and valid measurement instrument to help young people identify their career development competence is crucial for them to adapt to the challenging circumstances under the impacts of the pandemic and distinct social context.

### 1.3. Career-Related and Psychosocial Outcomes among Youth in the Transitional Status

In addition to the lack of research on YCDC measurement instruments under Hong Kong’s unique social context, research on the concurrent validity of YCDC measurement instruments is also scarce. The available literature indicates that YCDC may have positive relationships with career-related outcomes [41,42,43]. For instance, previous research has revealed that career adaptability—an individual’s resources to facilitate the successful management of current and anticipated career transitions—and career development competence are related [44]. Moreover, career outcome expectancy—an individual’s convictions concerning the probabilities of experiencing the results (e.g., earnings, self-satisfaction, and admiration) of their chosen career—were found to be strongly associated with career exploration intention [45], which is an important dimension of career development competence. Additionally, previous studies have revealed that career development competence may enhance one’s psychosocial outcomes [29,44]. For example, past studies have found that having a career and participating in career development activities promote social integration—a sense of belonging to a group or society [43,46]. In this sense, career development competence is expected to correlate with social integration. Furthermore, previous research has found that career-related service-learning activities enhance civic engagement—participation in activities for the common good [47]. This empirical observation implies that there may be a positive relationship between civic engagement and career development competence. In addition, prior studies have found that the contribution of volunteering facilitates student career commitment [48,49], which indicates an association between social contribution—deeds for the benefits of society—and career development competence. However, validation of the concurrent validity of YCDC measurement instruments is lacking in the literature. The concurrent validity of the YCDC scale developed in this study is, therefore, an important concern, involving correlations between YCDC and career-related and psychosocial outcomes.

### 1.4. Subgroup Consistency of the YCDC Scale

Additionally, the subgroup consistency validation of YCDC measurement instruments is also lacking in the literature. Due to different characteristics between subgroups, the stability of the measurement instrument may vary, and examination of such differences can enrich the literature. The validity of the YCDC scale developed in this study applies to the subgroups based on gender, age, and years of residence in Hong Kong [50,51,52]. Despite the possible difference in YCDC manifestations across different gender, age, and residence-year subsamples, previous research on YCDC scales lacks investigation of subgroup consistency. Accordingly, there is a need to examine subgroup consistency when developing the YCDC scale to ascertain whether it maintains a good model fit in different subgroups.

## 2. Materials and Methods

### 2.1. Procedure and Participants

Since the purpose of this study was to develop and validate a YCDC scale, the study recruited a youth sample of 682 respondents aged 13–29 (*M* = 19.52, *SD* = 3.29) who have participated in a territory-wide project titled CLAP@JC, which aims at empowering young people to become active path navigators and change agents of their career and life development pathways. The participants were informed about the purpose and procedures of the study. Parental consent was also obtained for participants under the age of 18. The method employed in this study was assessed and approved by an ethical review committee before administration.

Of the 682 participants, 56.7% were male. Most of the participants (80.9%) were Chinese, whereas 19.1% of them were ethnic minorities of South or Southeast Asian decent (e.g., Pakistani, Indonesians, and Filipinos). The majority of them (80.1%) were born in Hong Kong, and the median of their years of residence in Hong Kong was 17 years. A total of 14.3% of the participants were receiving public assistance provided by the government. Regarding their education level, the majority (56.9%) had received senior secondary level. As for their employment status, 35.4% were students, 33.5% were unemployed, 24.2% were in regular employment, 3.8% were self-employed or temporarily employed, and 3.1% were homemakers.

### 2.2. Measures

The development and validation of the structured questionnaire employed in this study included two parts. The first part consisted of the YCDC scale that was developed by the research team after making reference to the existing literature [10,24,26,27]. A total of 17 items were devised to measure four potential components, namely engagement, self-understanding, career and pathway exploration, and planning and career management. Six researchers and ten social workers with experience in the youth service field were invited to proofread and refine the scale to ensure its face validity. Subsequently, a pilot study was conducted with 14 young people to review the clarity of the questionnaire items. The second part included measures of career-related and psychosocial outcomes (i.e., career adaptability, career outcome expectancy, civic engagement, social contribution, and social integration), which were adopted from previous research conducted in different contexts [41,43,53] to check the concurrent validity of the YCDC scale.

#### 2.2.1. YCDC-Potential Components

*Engagement* refers to the ability of young people to get involved in new experiences, extend their connection with others and the community, and maintain an interest in participating in career and life planning activities [22]. This study developed four items to assess engagement by asking participants about their ability to do a list of things over the past month. Sample items included “Understand my competence and interests through participating in activities” and “Continuously participate in my selected activities and new experiences.” Each item was measured on a 5-point Likert scale from “1 = not confident at all” to “5 = highly confident.” Higher scores showed a higher level of engagement.

*Self-understanding* refers to the ability of young people to comprehend their hopes and dreams about work and life, engage in self-reflection and self-enhancement activities, and connect self-knowledge with pathway alternatives [22]. This study developed four items to assess self-understanding by asking participants about their ability to do a list of things over the past month. Sample items included “Maintain a sense of hope in achieving career and life development aspirations and goals” and “Verify my interests, competences, and values through daily life self-observations.” Each item was measured on a 5-point Likert scale from “1 = not confident at all” to “5 = highly confident.” Higher scores showed a higher level of self-understanding.

*Career and pathway exploration* refers to the ability to explore multiple pathways and multiple forms of work and career, compare pathway alternatives and prioritize them, as well as set career and life goals [22]. This study developed five items to assess career and pathway exploration by asking participants about their ability to perform a list of things over the past month. Sample items included “State learning and training approaches that equip me to achieve career and life development” and “Compare different career and life development pathways according to personal and environmental factors.” Each item was measured on a 5-point Likert scale from “1 = not confident at all” to “5 = highly confident.” Higher scores showed a higher level of career and pathway exploration.

*Planning and career management* refers to the ability to seek support and opportunities, overcome obstacles, and manage career transition and development [22]. This study developed four items to assess planning and career management by asking participants about their ability to do a list of things over the past month. Sample items included “Use self-management skills (e.g., interpersonal skills, time management, dependability, honesty, and problem-solving ability) to facilitate my performance and development in the workplace” and “Continuously develop my competences, interests, values, and understanding of the work world.” Each item was measured on a 5-point Likert scale from “1 = not confident at all” to “5 = highly confident.” Higher scores showed a higher level of planning and career management.

#### 2.2.2. Career Outcome Expectancy

Career outcome expectancy refers to young people’s convictions concerning the probabilities of experiencing the results of their chosen career [45,54]. It was assessed using five items adapted from the Vocational Outcome Expectancy Scale [41]. The participants were asked about their expectations of employment over the past month. Sample items included “Helps me to change lifestyle” and “Brings desirable salaries and benefits.” Each item was measured on a 5-point Likert scale from “1 = very low” to “5 = very high.” Higher scores showed a higher level of career outcome expectancy. The Cronbach’s alpha of this scale was 0.74.

#### 2.2.3. Career Adaptability

Career adaptability refers to young people’s resources that facilitate successful management of current and anticipated career transitions [18]. It was assessed by using 12 items adapted from the Career Adapt-Abilities Scale [43]. Participants were invited to rate the degree of their development of a list of abilities over the past month. Sample items included “Thinking about what my future will be like” and “Preparing for the future.” Each item was measured on a 5-point Likert scale from “1 = not strong” to “5 = very strong.” Higher scores showed a higher level of career adaptability. The Cronbach’s alpha of this scale was 0.92.

#### 2.2.4. Civic Engagement

Civic engagement refers to young people’s participation in activities for the common good [53]. Civic engagement was assessed using six items adapted from the Civic Engagement Scale [53]. Participants were invited to rate their level of civic engagement over the past month. Sample items included “Participated in voluntary work” and “Devoted efforts to my community.” Each item was measured on a 5-point Likert scale from “1 = never” to “5 = always.” Higher scores showed a higher level of civic engagement. The Cronbach’s alpha of this scale was 0.96.

#### 2.2.5. Social Contribution

Social contribution refers to young people’s deeds for the benefit of society [53]. The social contribution was assessed using five items adapted from the Social Contribution Scale [53]. Participants were invited to rate their level of social contribution over the past month. Sample items included “Did something beneficial for the community” and “Contributed something important to society.” Each item was measured on a 5-point Likert scale from “1 = never” to “5 = always”. Higher scores showed a higher level of social contribution. The Cronbach’s alpha of this scale was 0.95.

#### 2.2.6. Social Integration

Social integration refers to young people’s sense of belonging to a group or society [53]. Social integration was assessed using five items adapted from the Social Integration Scale [53]. Participants were invited to rate their level of social integration over the past month. Sample items included “Felt lonely and isolated” and “Felt accepted by my friends.” Each item was measured on a 5-point Likert scale from “1 = never” to “5 = always.” Higher scores showed a higher level of social integration. The Cronbach’s alpha of this scale was 0.73.

### 2.3. Data Analysis

Details of the analytic plans are presented in the following section.

*Exploratory factor analysis (EFA)*. The sample was randomly divided into two subsamples. One subsample (*n* = 343) was employed to conduct an EFA. A principal component analysis (PCA) was carried out on the data to decide the factor structure. The eigenvalues of the factors were set to be at least 1 [55], and varimax rotation was performed during the PCA. For factorability of the data, the Kaiser-Mayer-Olkin (KMO) measured the sampling adequacy and the Barlett test was employed. A value of KMO above 0.60 and significant test statistics from the Barlett test indicate that the data were suitable for factor analysis [56]. SPSS 26 (IBM Corp., Armonk, NY, USA) was used for EFA analysis.

*Confirmatory factor analysis (CFA).* Based on the results obtained in the EFA procedures, CFA was conducted using Mplus 8.2 [57] on the second randomly generated subsample (*n* = 339) to determine whether the model data fit between the item-factor structures obtained from the PCA. The evaluation of fit was based on the comparative fit index (CFI) [58], Tucker-Lewis index (TLI) [59], the root mean square error of approximation (RMSEA) [60], and standardized root mean square residual (SRMR) [61]. Values above 0.90 for the model fit index for CFI and TLI [58], 0.08 and below for SRMR [61], and 0.08 and below for RMSEA [62] are usually accepted as an acceptable fit.

*Subgroup consistency validation.* To further evaluate subgroup consistency for validating the stability of the scale, CFA was conducted for three respective pairs of subgroups: male vs. female; age ≥19 years (older) vs. age <19 years (younger), as individuals aged 10 to 19 are considered adolescents (World Health Organization, 2019); residence in Hong Kong >17 years (longer) and residence in Hong Kong ≤17 years (shorter), using the median as a cut-off for years of residence in Hong Kong [63].

*Internal consistency reliability.* The reliability of the YCDC scale and its subscales was evaluated according to the four-factor structure as reported in the factor analyses outlined above. Cronbach’s alpha coefficients were used for this examination, with 0.70 as the cutoff for acceptable reliability [64].

*Concurrent validity.* It was hypothesized that the YCDC would be significantly correlated with career-related outcomes (i.e., career adaptability and career outcome expectancy) [41,43], and psychosocial outcomes (i.e., civic engagement, social contribution, and social integration) [53] as found in the literature. Concurrent validity was evaluated through exploring the Pearson’s correlation coefficients between the YCDC scale as well as its subscales and the measures of career-related and psychosocial outcomes.

## 3. Results

### 3.1. Exploratory Factor Analysis

The EFA analysis results revealed that the KMO coefficient was 0.96 and the Chi-square from the Barlett test was 5085.61 (*p <* 0.001) for the YCDC scale. This result suggested that the data were suitable for the PCA. The PCA concluded that the YCDC scale had a four-factor structure accounting for 78.95% of the total variance. The first factor, “engagement,” consisted of four items. Factor loadings of the items included in this factor ranged between 0.56 and 0.95, accounting for 61.24% of the total variance in the scale. The second factor, “self-understanding,” consisted of four items. Factor loadings of the terms included in this factor ranged between 0.81 and 0.93, accounting for 7.08% of the total variance in the scale. The third factor, “career and pathway exploration,” consisted of five items. The loadings of the items included in this factor ranged between 0.77 and 0.87, accounting for 6.07% of the total variance in the scale. The fourth factor, “planning and career management”, consisted of 4 items. The loadings of the items included in this factor ranged between 0.70 and 0.90, accounting for 4.56% of the total variance in the scale. The contents and factor loadings of the items in the scale are presented in Table 1.

### 3.2. Confirmatory Factor Analysis

**Figure 1:**
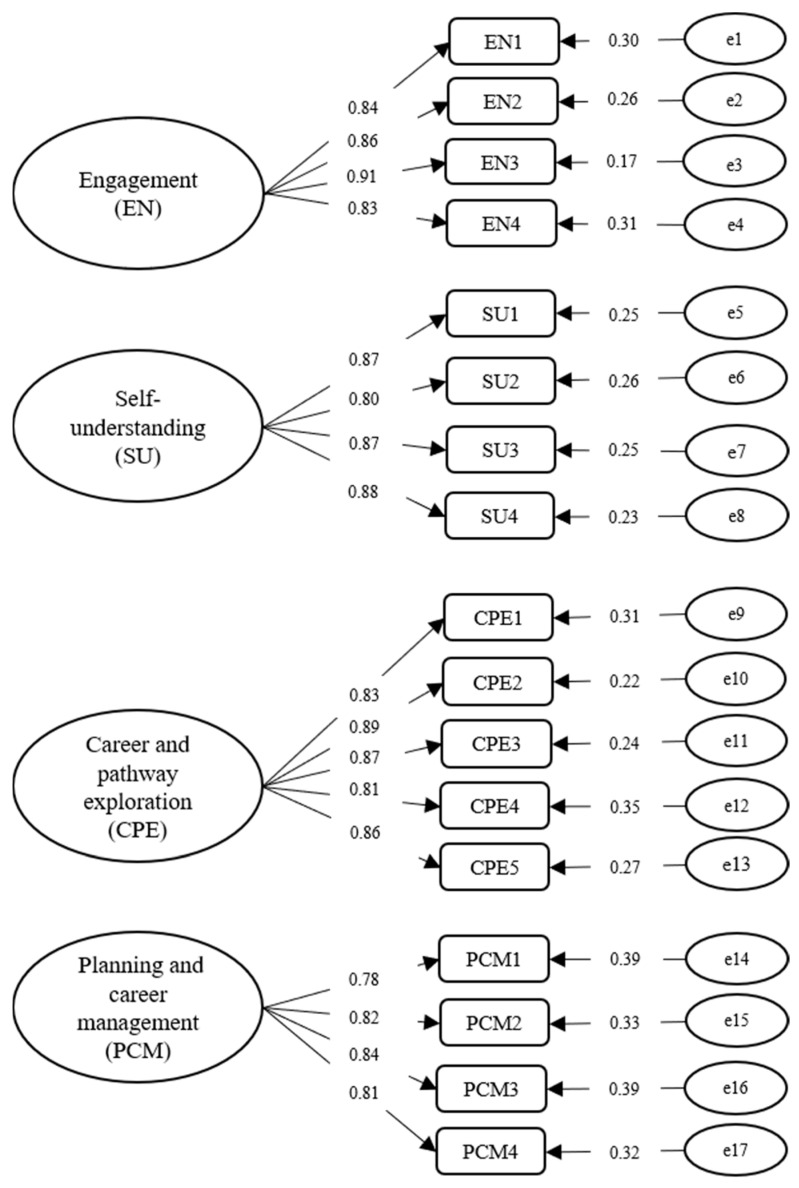
CFA factor structure and the standardized factor loadings (*n* = 339). All coefficients displayed in this figure are factor loadings that are statistically significant at *p <* 0.001 level.

The fit indices for the model obtained through the CFA were studied, and the Chi-square value was found to be significant (*χ^2^* = 258.48, *df* = 113, *χ^2^/df* = 2.29). The fit index values were found to be as follows: CFI = 0.96, TLI = 0.95, RMSEA = 0.06, 90% CI (0.05, 0.07), SRMR = 0.03. The fit index values suggest that the four-factor model yielded a good fit. The factor loadings concerning the four-dimensional model are presented in Figure 1. As can be concluded from Figure 1, the factor loadings ranged from 0.83 to 0.91 for engagement, from 0.80 to 0.88 for self-understand, from 0.81 to 0.89 for career and pathway exploration, and from 0.78 to 0.84 for planning and career management.

### 3.3. Reliability Estimation

An item analysis was conducted to determine the item-total correlation of the YCDC scale. The internal consistency of the scale and each subscale was estimated using Cronbach’s alpha. The analysis concluded that the corrected item-total correlations for the engagement, self-understanding, career and pathway exploration, and planning and career management subscales ranged from 0.69 to 0.77, 0.77 to 0.78, 0.73 to 0.79, and 0.68 to 0.75, respectively. The results revealed a high degree of homogeneity among the items within each subscale. The Cronbach’s alpha for engagement, self-understanding, career and pathway exploration, and planning and career management subscales was 0.92, 0.93, 0.93, and 0.89, respectively. The Cronbach’s alpha for the total scale was 0.96. The results indicated a satisfactory internal consistency, indicating that all subscales and the entire YCDC scale had good reliability. The results are presented in Table 2.

### 3.4. Factorial Validation in Subsamples

CFA studies were conducted separately across subsamples categorized by gender, age, and years of residence in Hong Kong. Goodness of fit indexed in the CFA is presented in Table 3. Satisfactory results were obtained in the following subsamples: male (*χ^2^* = 226.01, *df* = 113, *p <* 0.001, CFI = 0.97, RMSEA = 0.05, SRMR = 0.03) and female (*χ^2^* = 231.09, *df* = 113, *p <* 0.001, CFI = 0.96, RMSEA = 0.06, SRMR = 0.04); younger (*χ^2^* = 204.72, *df* = 113, *p <* 0.001, CFI = 0.97, RMSEA = 0.05, SRMR = 0.04) and older (*χ^2^* = 206.76, *df* = 113, *p <* 0.001, CFI = 0.97, RMSEA = 0.05, SRMR = 0.03); longer years of residence (*χ^2^* = 179.08, *df* = 113, *p <* 0.001, CFI = 0.98, RMSEA = 0.04, SRMR = 0.03) and shorter years of residence (*χ^2^* = 222.52, *df* = 113, *p <* 0.001, CFI = 0.96, RMSEA = 0.06, SRMR = 0.04).

### 3.5. Concurrent Validity

As mentioned earlier, career-related outcomes—career adaptability and career outcome expectancy [41,43]—and psychosocial outcomes—civic engagement, social contribution, and social integration [53]—were used to determine the concurrent validity of the YCDC scale. The correlation coefficients between the YCDC scale, the YCDC subscales, career adaptability, career outcome expectancy, civic engagement, social contribution, and social integration were calculated for verifying the concurrent validity of the scale. All the significant correlations between the variables showed good concurrent validity of the scale (see Table 4). As expected, the correlation coefficients between YCDC and career adaptability, career outcome expectancy, civic engagement, social contribution, and social integration were *r* = 0.70, *p <* 0.001; *r =* 0.37, *p <* 0.001; *r* = 0.42, *p <* 0.001; *r* = 0.44, *p <* 0.001; and *r* = 0.40, *p <* 0.001, respectively. Furthermore, the correlation coefficients between career adaptability and the sub-dimensions of YCDC were *r* = 0.59, *p <* 0.001; *r* = 0.63, *p <* 0.001; *r* = 0.60, *p <* 0.001; and *r* = 0.64, *p <* 0.001 for engagement, self-understanding, career and pathway exploration, and planning and career management, respectively. The correlation coefficients between career outcome expectancy and the sub-dimensions of YCDC were *r* = 0.33, *p <* 0.001; *r* = 0.34, *p <* 0.001; *r* = 0.33, *p <* 0.001; and *r* = 0.30, *p <* 0.001 for engagement, self-understanding, career and pathway exploration, and planning and career management, respectively. The correlation coefficients between civic engagement and the sub-dimensions of YCDC were *r* = 0.41, *p <* 0.001; *r* = 0.33, *p <* 0.001; *r* = 0.40, *p <* 0.001; and *r* = 0.34, *p <* 0.001 for engagement, self-understanding, career and pathway exploration, and planning and career management, respectively. The correlation coefficients between social contribution and the sub-dimensions of YCDC were *r* = 0.42, *p <* 0.001; *r* = 0.37, *p <* 0.001; *r* = 0.41, *p <* 0.001; and *r* = 0.36, *p <* 0.001 for engagement, self-understanding, career and pathway exploration, and planning and career management, respectively. The correlation coefficients between social integration and the sub-dimensions of YCDC were *r* = 0.40, *p <* 0.001; *r* = 0.39, *p <* 0.001; *r* = 0.36, *p <* 0.001; and *r* = 0.41, *p <* 0.001 for engagement, self-understanding, career and pathway exploration, and planning and career management, respectively.

## 4. Conclusions and Discussion

The findings of the present paper complement previous studies mainly from two perspectives. First, the literature in the field of YCDC measurement instruments has attached significance to three factors (i.e., self-understanding, career and pathway exploration, planning and career management) of career development competence for productive and meaningful school-to-work transitions [10,11,21,22]. This study attempted to contribute to the literature by incorporating an important yet often neglected factor—engagement—to build a four-factor scale to measure YCDC in the Hong Kong context. The EFA analysis results revealed that engagement accounted for the majority of variances in the total scale (i.e., 61.24%), which indicates its striking importance in YCDC. Moreover, as mentioned in the introduction, engagement is important for igniting, or re-igniting, young people’s motivation and enthusiasm through involvement in new significant career and life experiences, as well as their establishment of connections with supportive social networks during the career and life development journey. In other words, engagement enables young people to become active path navigators while developing interactive and mutually supportive relationships with parents, teachers, and the community to ensure a successful school-to-work transition [16].

Second, the collectivistic culture and family orientation in Chinese society always hinder young people’s development of self-understanding and self-exploration regarding their career aspirations [31,32,33,34]. For illustration, young people may make career decisions to meet the expectations of their parents instead of pursuing their own career interests. Indeed, the development and implementation of the YCDC scale may inspire young people’s awareness of personal agency and encourage them to initiate a self-guided career and life journey through identifying their career development competence. Therefore, incorporating engagement, self-understanding, career and pathway exploration, as well as planning and career management into the YCDC scale has significant theoretical and practical contributions to the field of young people’s career and life development. In addition, concurrent validity and subgroup consistency of YCDC measurement instruments, which have been overlooked in previous studies, were examined to further confirm that the developed scale was valid and stable.

The results of this study provide support for the applicability of the YCDC scale among young people in Hong Kong. Specifically, a principal component analysis was carried out on the 17-item YCDC scale, suggesting a four-factor structure, namely engagement, self-understanding, career and pathway exploration, and planning and career management. The four-factor structure accounts for 78.95% of the total variance in the YCDC scale. CFA was then conducted, and the results of CFA supported the multidimensional constructs obtained from EFA. Furthermore, the CFA results indicated satisfactory fit indices (CFI = 0.96, TLI = 0.95, RMSEA = 0.06, 90% CI (0.05, 0.07), SRMR = 0.03), and all the items in each subscale significantly represented their corresponding sub-constructs, which supported the construct validity of the YCDC scale. In addition, the internal consistency was calculated to determine the reliability of the YCDC scale. The reliability of the total scale was 0.96, and the subscales ranged between 0.89 and 0.93, indicating good reliability of the YCDC scale, which qualifies for the standard that the reliability of a scale is sufficient if its Cronbach’s alpha exceeds 0.70 [64].

Furthermore, subgroup consistency across gender, age, and residence-year subsamples was conducted. The results of CFA show a sufficient fit across three respective pairs of sub-groups: male and female, younger and older, shorter years of residence and longer years of residence. The results support subgroup consistency and further indicate that the YCDC scale is a promising measurement tool. Moreover, the results revealed that the YCDC scale and its subscales were positively correlated with career-related and psychosocial outcomes (i.e., career adaptability, career outcome expectancy, civic engagement, social contribution, and social integration). This aligns with the literature that has reported that career development competence is positively correlated with career adaptability [65], career outcome expectancy [45], civic engagement [47], social contribution [48], and social integration [43]. The significant associations with the expected directions in this study support the good concurrent validity of the YCDC scale. All the results demonstrate that the scale possesses adequate dimensions to assess the career development competence of young people in school-to-work transitions in the Chinese context of Hong Kong society.

Overall, it was demonstrated that the YCDC scale showed adequate reliability and satisfactory validity. Moreover, these results point to the value of YCDC in career-related research and interventions. On a theoretical level, engagement is incorporated in the measurement, and it will make great contributions to the measuring power of the YCDC to conduct various studies through the scale. In addition, given the current unpredictable working environment and multiple pathways of school-to-work transitions across the world, validation of the YCDC scale in another cultural context will further illustrate the cross-context applicability of this scale and help meet the imperative need for the development of a standardized instrument for measuring young people’s career development competence in school-to-work transitions.

On a practical level, the development of the YCDC scale will benefit policymakers, teachers, career counselors, and social workers. Strategies such as “Life Planning Education and Career Guidance for Secondary Schools” and “Task Force on Promotion of Vocational Education” to increase public awareness and promote vocational education and training in Hong Kong are launched by the government [66] to prepare diverse youth participants for productive school-to-work transitions [67]. Although these strategies are timely and commendable, they tend to attach importance to promotion and information sharing [3]. Less is mentioned about how to help those in school-to-work transitions identify their career development competence. Furthermore, while Hong Kong has some policies or guidelines for vocational education, the implementation of these policy mandates and guidelines varies and tends to merely become school-based curriculum programs. As a result, they fail to establish a clear articulation between study opportunities and career choices [2]. In this sense, developing a reliable and valid measurement instrument that can be used to help those youth in school-to-work transitions recognize their career development competence is essential. The YCDC scale developed in this study is warranted for policymakers, teachers, career counselors, and social workers to assist young people to identify their career development competence and successfully navigate transitions through education into employment.

Aside from the implications of the results, the results of this study should be viewed with some limitations on their generalizability. The sample of the study consisted of youth who have participated in the CLAP@JC project, and the generalizability of the results might be limited to the young people in the status of school-to-work transition in Hong Kong. Furthermore, the median was used as a cut-off point for the years of residence in Hong Kong in the subgroup analysis. Future studies may specifically examine the results of young people with more specific years of residence in Hong Kong. A second limitation concerns the sample size imbalance in educational levels (i.e., 56.9% received a senior secondary level, while only 2.9% received a bachelor’s or above), such that an education-related subgroup analysis was not possible in the present study due to the limited subsample sizes of those at different education levels. Future studies might consider recruiting participants at different educational levels to explore the capacity of the instrument in assessing YCDC of various education subgroups. In addition, because of the cross-sectional nature of the present study, it is necessary to verify the test–retest reliability of this scale by longitudinal data in future studies.

## Figures and Tables

**Table 1:** Rotated factor loadings matrix from EFA (*n* = 343).

Items	Factors			
	F1	F2	F3	F4
Understand my competences and interests through participating in activities.	0.93			
Continuously participate in my selected activities and new experiences.	0.95			
Participate in activities that are helpful to my career and life development.	0.88			
Understand the career and life development planning process and steps.	0.56			
Consider different career and life development pathway choices based on my attributes (e.g., interests).		0.93		
Maintain a sense of hope in achieving career and life development aspirations and goals.		0.81		
Verify my interests, competences, and values through daily life self-observations.		0.89		
Choose career and life development pathway and direction according to self-attributes (e.g., interests, competences).		0.85		
State learning and training approaches that equip me to achieve career and life development goals.			0.77	
Compare different career and life development pathways according to personal and environmental factors.			0.85	
Choose the most suitable career and life development pathway according to personal and environmental factors.			0.87	
Identify personal limitations and social barriers encountered when pursuing career and life development goals.			0.81	
Know the necessary steps in making good career and life development decisions and understand the strengths and limitations of my decision-making methods.			0.83	
Use self-management skills (e.g., interpersonal skills, teamwork, time management, dependability, honesty, and problem-solving ability) to facilitate my performance and development in the workplace.				0.90
Obtain relevant support and guidance to resolve difficulties related to career and life development in future.				0.84
Continuously develop my competences, interests, values and understanding of the work world.				0.70
Cope with future career and life development transitions and changes, as well as the stress involved.				0.81

Note: F1 = Engagement; F2 = Self-understanding; F3 = Career and Pathway Exploration; F4 = Planning and Career Management.

**Table 2:** Scale statistics and item-total correlations for the YCDC scale.

Subscale	Item	Scale Mean	Scale Variance	Item-Total Correlation	Cronbach’s Alpha for Subscale
Engagement (EN)	EN1	51.53	134.05	0.69	0.92
	EN2	51.50	134.04	0.69	
	EN3	51.51	132.54	0.73	
	EN4	51.56	132.50	0.77	
Self-understanding (SU)	SU1	51.41	133.80	0.77	0.93
	SU2	51.45	132.64	0.78	
	SU3	51.40	132.90	0.78	
	SU4	51.40	132.32	0.78	
Career and pathway exploration (CPE)	CPE1	51.71	132.95	0.77	0.93
	CPE2	51.64	133.55	0.78	
	CPE3	51.62	132.41	0.79	
	CPE4	51.62	133.77	0.73	
	CPE5	51.64	132.08	0.79	
Planning and career management (PCM)	PCM1	51.51	134.55	0.68	0.89
	PCM2	51.59	134.19	0.72	
	PCM3	51.47	133.04	0.75	
	PCM4	51.58	133.44	0.71	

**Table 3:** Factorial validation in subsamples categorized by gender, age, and years of residence in Hong Kong.

	CFA of Total Sample Model *N* = 682	Gender		Age		Residence in Hong Kong	
		Male *n* = 387	Female *n* = 295	Younger *n* = 308	Older *n* = 363	Longer *n* = 361	Shorter *n* = 314
Chi-square	325.51	226.01	231.09	204.72	206.76	179.08	222.52
Degrees of freedom	113	113	113	113	113	113	113
*p*-value	<0.001	<0.001	<0.001	<0.001	<0.001	<0.001	<0.001
CFI	0.97	0.97	0.96	0.97	0.97	0.98	0.96
RMSEA	0.05	0.05	0.06	0.05	0.05	0.04	0.06
SRMR	0.03	0.03	0.04	0.04	0.03	0.03	0.04

**Table 4:** Correlations between the YCDC scale and its subscales with career-related and psychosocial outcomes.

	1	2	3	4	5	6	7	8	9	10
1. YCDC	1.00									
2. EN	0.85 ***									
3. SU	0.89 ***	0.69 ***	1.00							
4. CPE	0.91 ***	0.67 ***	0.76 ***	1.00						
5. PCM	0.87 ***	0.63 ***	0.69 ***	0.76 ***	1.00					
6. CA	0.70 ***	0.59 ***	0.63 ***	0.60 ***	0.64 ***	1.00				
7. COE	0.37 ***	0.33 ***	0.34 ***	0.33 ***	0.30 ***	0.35 ***	1.00			
8. CE	0.42 ***	0.41 ***	0.33 ***	0.40 ***	0.34 ***	0.35 ***	0.25 ***	1.00		
9. SC	0.44 ***	0.42 ***	0.37 ***	0.41 ***	0.36 ***	0.36 ***	0.30 ***	0.83 ***	1.00	
10. SI	0.44 ***	0.40 ***	0.39 ***	0.36 ***	0.41 ***	0.43 ***	0.22 ***	0.17 ***	0.26 ***	1.00

Note: EN = Engagement; SU = Self-understanding; CPE = Career and Pathway Exploration; PCM = Planning and Career Management; CA = Career Adaptability; COE = Career Outcome Expectancy; CE = Civic Engagement; SC = Social Contribution; SI = Social Integration; *** *p* < 0.001.

## Data Availability

The datasets generated and/or analyzed in the current study are not publicly available, as they contain information that could compromise the privacy of research participants. The data that support the findings of this study are available from the corresponding author upon reasonable request.

## References

[1] Wells K.L.H. (2018). Laboring Under Globalization: Tapestries by Contemporary Artists. Art J..

[2] Pavlova M., Lee J.C.K., Maclean R. (2017). Complexities of School to Work Transitions. Educ. Res. Policy Pract..

[3] Pavlova M., Lee J.C.K., Maclean R. (2018). Transitions to Post-School Life: Responsiveness to Individual, Social and Economic Needs.

[4] European Centre for the Development of Vocational Training (2014). Navigating Difficult Waters: Learning for Career and Labour Market Transition.

[5] Koivisto P., Vuori J., Nykyri E. (2007). Effects of the School-to-Work Group Method among young people. J. Vocat. Behav..

[6] Koivisto P., Vuori J., Vinokur A.D. (2010). Transition to Work: Effects of Preparedness and Goal Construction on Employment and Depressive Symptoms. J. Adolesc. Res..

[7] Huegaerts K., Spruyt B., Vanroelen C. (2018). Youth Unemployment and Mental Health: The Mediating Role of Embodiment. Societies.

[8] Ngai S.S.Y., Cheung J.C.K., To S.M., Luan H., Zhao R. (2014). Economic Disadvantage and Transitional Outcomes: A Study of Young People from Low-income Families in Hong Kong. J. Youth Adolesc..

[9] Wong V. (2012). Social Withdrawal as Invisible Youth Disengagement: Government Inaction and NGO Responses in Hong Kong. Int. J. Sociol. Soc. Policy.

[10] Akkermans J., Brenninkmeijer V., Huibers M., Blonk R.W.B. (2013). Competencies for the Contemporary Career: Development and Preliminary Validation of the Career Competencies Questionnaire. J. Career Dev..

[11] Kuijpers M., Scheerens J. (2006). Career Competencies for the Modern Career. J. Career Dev..

[12] Segers J., Inceoglu I. (2012). Exploring Supportive and Developmental Career Management through Business Strategies and Coaching. Hum. Resour. Manag..

[13] Vuori J., Toppinen-Tanner S., Mutanen P. (2012). Effects of Resource-Building Group Intervention on Career Management and Mental Health in Work Organizations: Randomized Controlled Field Trial. J. Appl. Psychol..

[14] Zamfir A.-M., Militaru E., Mocanu C., Lungu E.O. (2020). School-to-Work Transition of Higher Education Graduates in Four European Countries. Compare.

[15] Raffe D. (2008). The Concept of Transition System. J. Educ. Work.

[16] Mann A., Denis V., Percy C. (2020). Career Ready? How Schools Can Better Prepare Young People for Working Life in the Era of COVID-19.

[17] De Vos A., De Clippeleer I., Dewilde T. (2009). Proactive Career Behaviours and Career Success During the Early Career: Careers Research in Europe: Identity and Contribution. J. Occup. Organ. Psychol..

[18] Savickas M.L., Brown S.D., Lent R.W. (2005). The Theory and Practice of Career Construction. Career Development and Counseling: Putting Theory and Research to Work.

[19] Briscoe J.P., Hall D.T., Frautschy DeMuth R.L. (2006). Protean and Boundaryless Careers: An Empirical Exploration. J. Vocat. Behav..

[20] Briscoe J.P., Hall D.T. (2006). The Interplay of Boundaryless and Protean Careers: Combinations and Implications. J. Vocat. Behav..

[21] Anakwe U.P., Hall J.C., Schor S.M. (2000). Knowledge-related Skills and Effective Career Management. Int. J. Manpow..

[22] CLAP for Youth@JC (2017). Report for Mid-Term Review.

[23] Kuijpers M., Meijers F., Gundy C. (2011). The Relationship between Learning Environment and Career Competencies of Students in Vocational Education. J. Vocat. Behav..

[24] Kuijpers M.A.C.T., Schyns B., Scheerens J. (2006). Career Competencies for Career Success. Career Dev. Q..

[25] Kossek E.E., Roberts K., Fisher S., Demarr B. (1998). Career Self-management: A Quasi-experimental Assessment of the Effects of a Training Intervention. Pers. Psychol..

[26] Francis-Smythe J., Haase S., Thomas E., Steele C. (2013). Development and Validation of the Career Competencies Indicator (CCI). J. Career Assess..

[27] Son Y., Lim E., Song C., Bang H., Youn S., Kim H., Lee Y. (2015). 2015 Career Education Center: School Career Education Program System.

[28] Saks A.M. (2006). Antecedents and Consequences of Employee Engagement. J. Manag. Psychol..

[29] Bakker A.B., Demerouti E. (2017). Job Demands-Resources Theory: Taking Stock and Looking Forward. J. Occup. Health Psychol..

[30] Census and Statistics Department (2020). Quarterly Report on General Household Survey.

[31] International Labor Organization (2019). Asia-Pacific Employment and Social Outlook: Advancing Decent Work for Sustainable Development.

[32] Boys’ and Girls’ Clubs Association of Hong Kong (2015). Research Results on Life Planning and Career Choices of Secondary Six Students in Hong Kong. https://www.bgca.org.hk/en-us/article/237.

[33] Cheung F.M., van de Vijver F.J.R., Leong F.T.L. (2011). Toward a New Approach to the Study of Personality in Culture. Am. Psychol..

[34] Arnold J. (2014). The Impact of Career Exploration on Career Development Among Hong Kong Chinese University Students. J. Coll. Stud. Dev..

[35] Leung S.A. (2002). Career Counseling in Hong Kong: Meeting the Social Challenges. Career Dev. Q..

[36] Fan W., Cheung F.M., Leong F.T.L., Cheung S.F. (2014). Contributions of Family Factors to Career Readiness: A Cross-Cultural Comparison. Career Dev. Q..

[37] Leung S.A., Hou Z.-J., Gati I., Li X. (2011). Effects of Parental Expectations and Cultural-values Orientation on Career Decision-making Difficulties of Chinese University Students. J. Vocat. Behav..

[38] Guo K. (2013). Ideals and Realities in Chinese Immigrant Parenting: Tiger Mother Versus Others. J. Fam. Stud..

[39] Cheng S., Yuen M. (2012). Validation of the Career-Related Parent Support Scale Among Chinese High School Students. Career Dev. Q..

[40] Hui T., Yuen M., Chen G. (2018). Career-Related Filial Piety and Career Adaptability in Hong Kong University Students. Career Dev. Q..

[41] Iwanaga K., Umucu E., Wu J.-R., Yaghmaian R., Lee H.L., Fitzgerald S., Chan F. (2020). Assessing Vocational Outcome Expectancy in Individuals with Serious Mental Illness: A Factor-analytic Approach. J. Ment. Health.

[42] Mascherini M., Salvatore L., Meierkord A., Jungblut J.M. (2012). NEETs: Young People Not in Employment, Education or Training: Characteristics, Costs and Policy Responses in Europe.

[43] Savickas M.L., Porfeli E.J. (2012). Career Adapt-Abilities Scale: Construction, Reliability, and Measurement Equivalence across 13 Countries. J. Vocat. Behav..

[44] Akkermans J., Paradniké K., Van der Heijden B.I., De Vos A. (2018). The Best of Both Worlds: The Role of Career Adaptability and Career Competencies in Students’ Well-Being and Performance. Front. Psychol..

[45] Diegelman N.M., Subich L.M. (2001). Academic and Vocational Interests as a Function of Outcome Expectancies in Social Cognitive Career Theory. J. Vocat. Behav..

[46] Siddique C.M. (1981). Orderly Careers and Social Integration. Ind. Relat..

[47] Ma C.H.K., Chan C.W.F., Chan A.C.M. (2016). The Long-term Impact of Service-learning on Graduates’ Civic Engagement and Career Exploration in Hong Kong. J. High. Educ. Outreach Engagem..

[48] Cheung C.K., Liu E.S.C. (2017). Enhancing the Contribution of Volunteering to Career Commitment with Friendship among University Students. Career Dev. Int..

[49] Coulter-Kern R.G., Coulter-Kern P.E., Schenkel A.A., Walker D.R., Fogle K.L. (2013). Improving Student’s Understanding of Career Decision-Making through Service Learning. J. Coll. Stud. Dev..

[50] Bocciardi F., Caputo A., Fregonese C., Langher V., Sartori R. (2017). Career Adaptability as a Strategic Competence for Career Development: An Exploratory Study of Its Key Predictors. Eur. J. Train. Dev..

[51] Rohlfing J.E., Nota L., Ferrari L., Soresi S., Tracey T.J.G. (2012). Relation of Occupational Knowledge to Career Interests and Competence Perceptions in Italian Children. J. Vocat. Behav..

[52] Zacher H. (2014). Individual Difference Predictors of Change in Career Adaptability over Time. J. Vocat. Behav..

[53] Ngai S.S.Y., Cheung C.K., Chan C.T. (2017). The Interim Report of the Cyber Youth Outreach: A Project on ICT-Enhanced Initiatives for Youth at Risk of Social Exclusion.

[54] Lent R.W., Brown S.D., Hackett G. (1994). Toward a Unifying Social Cognitive Theory of Career and Academic Interest, Choice, and Performance. J. Vocat. Behav..

[55] Shevlin M.E., Lewis C.A. (1999). The Revised Social Anxiety Scale: Exploratory and Confirmatory Factor Analysis. J. Soc. Psychol..

[56] Tabachnick B.G., Fidell L.S. (2013). Using Multivariate Statistics: Pearson New International Edition.

[57] Muthén B., Muthén L., van der Linden W.J. (2017). Mplus. Handbook of Item Response Theory, Volume Three: Applications.

[58] Hu L.T., Bentler P.M. (1999). Cutoff Criteria for Fit Indexes in Covariance Structure Analysis: Conventional Criteria Versus New Alternatives. Struct. Equ. Modeling.

[59] Tucker L.R., Lewis C. (1973). A Reliability Coefficient for Maximum Likelihood Factor Analysis. Psychometrika.

[60] Steiger J.H. (1990). Structural Model Evaluation and Modification: An Interval Estimation Approach. Multivar. Behav. Res..

[61] Schumacker R.E., Lomax R.G. (2010). A Beginner’s Guide to Structural Equation Modeling.

[62] Cole D.A. (1987). Utility of Confirmatory Factor Analysis in Test Validation Research. J. Consult. Clin. Psychol..

[63] DeCoster J., Gallucci M., Iselin A.M.R. (2011). Best Practices for Using Median Splits, Artificial Categorization, and their Continuous Alternatives. J. Exp. Psychopathol..

[64] Pallant J. (2020). SPSS Survival Manual: A Step by Step Guide to Data Analysis Using IBM SPSS.

[65] Akkermans J., Tims M. (2017). Crafting your Career: How Career Competencies Relate to Career Success via Job Crafting. Appl. Psychol..

[66] Information Services Department The Facts Hong Kong as a Service Economy.

[67] Education Bureau (2014). Education Bureau Circular No. 1/2009: Upholding Students’ Right to Education.

